# A refined phenomenological model of viscoelastic clot formation and lysis in trauma-induced coagulopathy

**DOI:** 10.3389/fcvm.2026.1816085

**Published:** 2026-09-03

**Authors:** Guanyun Liu, Amanda E. Shick, Maya Shamash, Caroline M. Cook, Mario L. Molina, Mitchell J. Cohen, Amor A. Menezes

**Affiliations:** 1 Department of Mechanical and Aerospace Engineering, University of Florida, Gainesville, FL, United States; 2 Department of Agricultural and Biological Engineering, University of Florida, Gainesville, FL, United States; 3 Department of Surgery, University of Colorado Anschutz Medical Campus, Aurora, CO, United States; 4 J. Crayton Pruitt Family Department of Biomedical Engineering, University of Florida, Gainesville, FL, United States; 5 Genetics Institute, University of Florida, Gainesville, FL, United States; 6 Department of Chemical Engineering, University of Florida, Gainesville, FL, United States

**Keywords:** blood coagulation, cybermedicine, dynamical system model, hyperfibrinolysis, thromboelastography (TEG), tissue plasminogen activator (tPA), tranexamic acid (TXA), trauma-induced coagulopathy (TIC)

## Abstract

**Background:**

Hyperfibrinolysis is strongly associated with early post-injury mortality, but the condition is preventable with timely detection and targeted interventions. Unfortunately, current hyperfibrinolysis diagnostic assays such as thromboelastography (TEG) are time-consuming, making them infeasible for use in real-time treatment strategies to guide adjustable, precise, and personalized interventions. Computational models offer a fast alternative to current practice for ascertaining patient hemostatic state from quickly-measurable protein concentrations. However, few models exist to predict clot strength. Our prior biologically-interpretable, phenomenological, dynamical system TEG model does not often satisfactorily capture hyperfibrinolysis because this feature was missing from training data. Here, we present model improvements that are experimentally-driven and theoretically-grounded to better facilitate the replacement of slow patient viscoelastic clotting measurements, with and without hyperfibrinolysis, by rapid and accurate predictions of TEG parameters *in silico*.

**Methods:**

We created a tPATXA dataset to enable model refinements, and we validated TEG parameter predictions using the published Activation of Coagulation and Inflammation in Trauma (ACIT) dataset, which was excluded from model training. Our tPATXA dataset consists of 310 citrated native (CN) TEG assay profiles that were generated from healthy human donor whole blood samples spiked with tissue plasminogen activator (tPA) and tranexamic acid (TXA) at varying concentrations to provide added phenomenological information about fibrinolysis behavior. We then used ACIT clinical data from 93 trauma patients containing 254 citrated kaolin (CK) and 122 citrated kaolin with heparinase (CKH) TEG assays for model validation. We characterized model performance by: the coefficient of determination $R^{2}$; percent-error predictions of TEG parameters R-time, K-time, alpha angle, maximum amplitude, and time to maximum amplitude; and absolute errors of TEG parameters Ly30 and Ly60.

**Results:**

Our key model update is to make a previously-constant parameter a time-varying function. Our updated model substantially outperforms the prior model in both datasets. On the tPATXA dataset that both models were trained on and then subsequently predicted to verify competency, the model updates improved $R^{2}$ from 0.9726 to 0.9983. On the ACIT validation dataset that was not used for training, the model updates improved $R^{2}$ from 0.9848 to 0.9993, and reduced TEG parameter prediction variance by over 99%. In addition, the updated model had low prediction errors of 1%–13% for R-time, K-time, alpha angle, maximum amplitude, and time to maximum amplitude, and 0.7%–2.2% for Ly30 and Ly60. A mechanistic interpretation of the new parameter is its capture of the formation and breakdown of a fibrin clot mesh over time.

**Conclusions:**

Our refined model offers higher accuracy, consistency, and biological interpretability than previously available. Our updated model’s modular design supports future integration with literature phenomenological models that predict thrombin dynamics, as well as with externally-added controllers for automation. Thus, this work captures broad clinical coagulation insights and also lays the groundwork for real-time, personalized trauma care via control-theoretic tools.

## Introduction

Trauma has been the lead cause of death in the U.S. for ages 1–44 in every year from 1981–2024 [1]. In 2023, deaths from injuries and violence were more than seven times the second-leading cause of death [1]. The presence of shock puts severely injured patients at high risk for developing trauma-induced coagulopathy (TIC) [2], a condition that is characterized by abnormal blood clotting due to a disrupted balance between clot formation and degradation [3, 4]. TIC has multiple phenotypes that can be categorized as either hypocoagulation or hypercoagulation [4], depending on whether a patient exhibits hemorrhage (uncontrolled bleeding) or thrombosis (overclotting) symptoms, respectively. Patients can also experience mixed phenotypes, since TIC may gradually transition from initial hypocoagulopathy within the first six hours after injury to hypercoagulopathy in late TIC about 24 h after injury [4]. Among the various TIC hypocoagulation phenotypes, hyperfibrinolysis, which induces hemorrhage, is a well-known contributor to mortality [5, 6], and is a strong independent predictor of death [7] in early TIC. Although the exact pathophysiology remains unclear, hyperfibrinolysis occurs when the fibrinolytic process is overly activated by elevated levels of tissue plasminogen activator (tPA) [4, 8], leading to an excessive production of plasmin that destabilizes the blood clot and causes uncontrolled bleeding.

**Figure 1:**
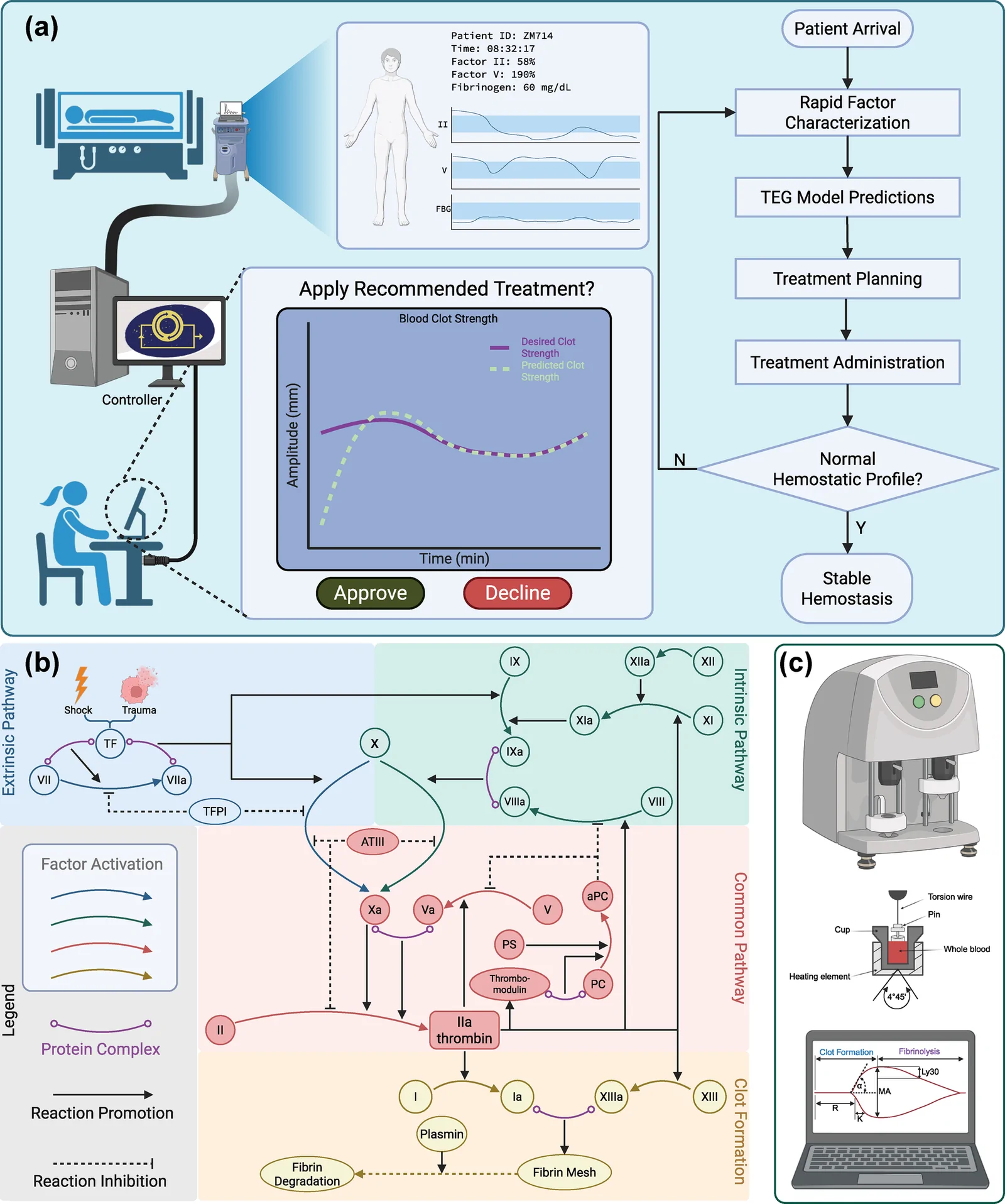
Overview of coagulation cybermedicine. **(a)** The envisioned cybermedical treatment system includes (1) a point-of-care sensor suite to frequently characterize patient hemostasis profile, (2) an advanced control algorithm that automatically and quickly suggests a treatment plan that is tailored to each patient, and (3) a user-friendly interface that allows clinicians to review, adjust, and approve the suggested treatment. The cybermedical treatment scheme is a core element of a clinical workflow, which has patient blood samples being collected upon hospital arrival and as frequently as possible thereafter, and therapeutic interventions being suggested and administered after every sample collection based on the values of sample measurements. The process of sample measurement and treatment suggestion is repeated until coagulopathy is remediated. **(b)** The coagulopathy cybermedical treatment scheme manipulates the concentrations of proteins, called coagulation factors, whose interactions make up the coagulation cascade, shown in simplified form here. **(c)** The coagulation cascade produces clots whose strength can be measured viscoelastically by a TEG5000 analyzer. A torsion wire measures the clotting resistance of whole blood that is placed in an oscillating, heated cup, to produce viscoelastic curves that describe clot dynamics.

Prompt detection of hyperfibrinolysis will reduce early trauma patient mortality, and detected hyperfibrinolysis can be addressed with personalized and targeted treatments to precisely regulate blood clot strength. We envision the use of a coagulation cybermedical system [9], Figure 1a, to provide personalized trauma care that is optimized for the best treatment outcome by leveraging iterative blood sample measurements when suggesting interventions whose administration will rectify trauma patient coagulation back to normal. This approach will modulate the concentrations of coagulation factors (blood proteins) that interact to form the coagulation cascade, Figure 1b. Thus, the cybermedical system will consist of sensors [10], a controller [11–13], and actuators [14]. The sensor suite will continuously (or very frequently) update a patient’s hemostasis profile from measured coagulation factor concentrations, allowing the controller to dynamically adjust actuator interventions such as coagulation factor levels in a blood transfusion unit based on differences between a patient’s current condition and a target clotting profile. It takes less than four minutes, on average, to measure the concentration of a single coagulation factor in a blood sample using the STA Compact Max (Stago) in our experience. While more rapid sensing of coagulation factor levels does not yet exist, related biosensing technologies to quickly quantify blood analytes have been reported [15, 16]. These technologies suggest that frequent sensing is feasible, although substantial development still remains. The cybermedical system will also have a user-friendly interface for clinicians to monitor patient coagulation status, treatment delivery, and therapeutic efficacy (both predicted and actual). For safety, clinicians will be able to approve or reject any cybermedical system intervention recommendations based on their expertise. This article improves the representations from an anticipated coagulation cybermedical system sensor suite to better guide intervention recommendations.

Thromboelastography (TEG) and Rotational Thromboelastometry (ROTEM) are two common assays that characterize the viscoelasticity of blood samples [17]. Figure 1c overviews the TEG5000 analyzer. This device has two channels and can thus characterize the viscoelasticity of two samples simultaneously. For each channel, a whole blood sample is placed in a heated measuring cup that is designed to maintain the sample at body temperature ($37{\;}^{\circ }\text{C}$). The cup is then raised into position, immersing a stationary pin. During the test, the cup continuously rotates $\pm 4^{\circ }45^{\prime }$, allowing the immersed pin to sense shear forces that are generated by clot formation and breakdown. A ROTEM device works similarly, except that it is the pin that rotates within a stationary cup. Both TEG and ROTEM measure and graph blood clot strength in real time, generating static metrics that provide insight into coagulation and fibrinolysis while plotting a time course viscoelastic trajectory.

Figure 1c includes a sample trajectory of amplitude vs. time that is produced by the TEG5000 analyzer. This trajectory contains two symmetric curves about the horizontal axis, labeled with key static TEG output parameters. The signs of the amplitude align with the direction of cup rotation. The output parameters are R-time, K-time, alpha angle, maximum amplitude, time to maximum amplitude, Ly30, and Ly60. The R-time (in minutes) is the time from the start of the test to the time at which the amplitude passes 1 mm, which indicates how quickly the blood sample starts to clot. The K-time (in minutes) is the time required for the clot strength to rise from 1 mm to 20 mm, which reflects the rate of clot formation. The alpha angle (in degrees) is the slope angle of the tangential line to the curve passing through the 1 mm amplitude, which suggests the rate of clot formation. The maximum amplitude (MA, in millimeters) of the TEG profile corresponds to the maximum clotting strength, and the time required to achieve this peak value is the time to maximum amplitude (TMA, in minutes). Ly30 and Ly60 measure the percentage of clot lysis at 30 min and 60 min, respectively, after TMA, which indicates the rate of clot breakdown. These parameters and overall trajectory allow clinicians to plan appropriate treatments, including fresh-frozen plasma transfusions [18], procoagulant treatments such as tranexamic acid (TXA) [19], and anticoagulant treatments such as heparin [20].

A single rapid TEG analysis can take at least 15 min to ascertain TEG parameters of R-time, K-time, alpha angle, and MA without any lysis parameters (Ly30 and Ly60) [21], while a complete citrated native (CN)-TEG run takes about 90 min. The typical time from sample collection to report is much longer for both rapid TEG and CN-TEG. Relying on experimental TEG assays to provide frequent updates of patient clotting status is infeasible in practice because repetitive TEG assays substantially burden the clinical care team and saddle patients with the extra costs of expensive reagents.

Another widely used coagulation assay is the Calibrated Automated Thrombogram (CAT) [22, 23]. The CAT measures the entire process of thrombin generation when the coagulation cascade clotting mechanism (simplified in Figure 1b) is triggered by an input of tissue factor. Like TEG and ROTEM, this measurement takes too much time to be useful in urgent care. A single CAT assay takes about 60 min to run, with no rapid version available.

Despite the use of TEG (or ROTEM) and CAT coagulation assays to guide resuscitation, it remains challenging to deliver personalized trauma care that can precisely determine the optimal combination and dosage of coagulation factors, inhibitors, and other hemostatic agents. First, current treatment plans [4] are largely protocol-driven rather than fully personalized. Such structured, predefined, evidence-based guidelines allow healthcare providers to avoid the worst medical care outcomes, but they also prevent the best, optimal ones [24]. Second, precise and targeted trauma care requires timely adjustment of treatment strategies based on the hemostasis profile of a patient. Ideally, treatment outcomes will be optimized if clinicians had real-time updates of a patient’s clotting status, but this is impractical given the time-consuming nature of coagulation assays. To provide interventions within the golden hour of trauma patient care [25, 26], clinicians have to follow protocol-driven treatments because it is impossible to collect a blood sample, wait for a TEG analysis, formulate a treatment plan, and prepare blood products in a timely manner.

Model-based *in silico* predictions may address the problem of long waiting times associated with experimental assays, not only helping to save time but also allowing clinicians to make patient-tailored medical decisions by providing more frequent assessments of patient condition. Apart from our prior work [9], few computational TEG models exist, and these alternate models [27, 28] do not take coagulation factor concentrations as their input. It is desirable to use coagulation factor concentrations as an input into a model that makes predictions of TEG curves since each concentration can be measured in an order-of-magnitude less time than a TEG assay. We previously [9] showed that a six-parameter time-invariant TEG model can replace CN-TEG assays to provide instant yet accurate predictions of a blood sample’s viscoelastic status, given input measurements of coagulation factor levels. As shown in Figure 1a, upon patient arrival, their coagulation factor levels will be rapidly characterized and then used to estimate TEG model parameters. Because factor levels vary from patient to patient, the model parameters will be patient-specific. Thereafter, our TEG model will instantaneously predict TEG profiles and the associated parameters (R-time, K-time, etc.). These predictions will inform treatment plan development and therapeutic administration, toward restoring normal hemostatic profiles.

However, our prior six-parameter time-invariant TEG model does not often satisfactorily capture hyperfibrinolysis because this feature was missing from training data. We therefore propose a nine-parameter time-varying TEG model that addresses this limitation and that can also provide accurate predictions of TEG parameters regardless of the type of TEG assay. Our updated model encompasses the following intellectual contributions:

1. Elimination of discontinuities while further reducing prediction error;
2. *In vitro* CN-TEG verification on healthy human donor whole blood samples treated in different ways to simulate fibrinolysis in trauma patient blood samples; and
3. Validation using actual trauma patient data to demonstrate improved model robustness.

## Materials and methods

### tPATXA dataset

We created a tPATXA dataset to enable model refinements. Thromboelastography (Haemonetics, TEG5000) was used to analyze single donor sodium citrated human whole blood samples (Cedarlane, IWB1NAC10ML) spiked with varying concentrations of tPA (Sigma Aldrich) and the antifibrinolytic agent TXA (Santa Cruz Biotechnology) reconstituted in sterile 1X PBS (Corning) and 5% bovine serum albumin (BSA, Fisher Bioreagents). Fifteen biological replicates were analyzed, with results averaged across two technical replicates. The tPA concentrations tested were 0, 50, 75, and 100 ng/mL, and the TXA concentrations tested were 0, 0.075, 0.25, 0.5, 1, 2, 4, 6, 8, 10, 50, 100, 150, and 200 μ g/mL. The tPA concentrations were varied to represent different degrees of hyperfibrinolysis, a Ly30 value greater than 7.5% being clinically described as hyperfibrinolytic. TXA concentrations were then varied to determine changes in clotting behavior given coagulopathy severity.

Standard TEG operating procedures were followed [29]. Briefly, 20 μ L of 0.2M CaCl${}_{2}$ and 340 μ L of room temperature citrated whole blood were added to a disposable cup secured in the TEG5000. The cup was moved into the start position and the TEG software was immediately started, using the citrated native (CN) test. The TEG was allowed to run to completion and then output data was analyzed. A total of 310 TEG assay profiles were created, of which, 276 had a complete set of TEG parameter and time-history data that were useful for model refinements.

**Figure 2:**
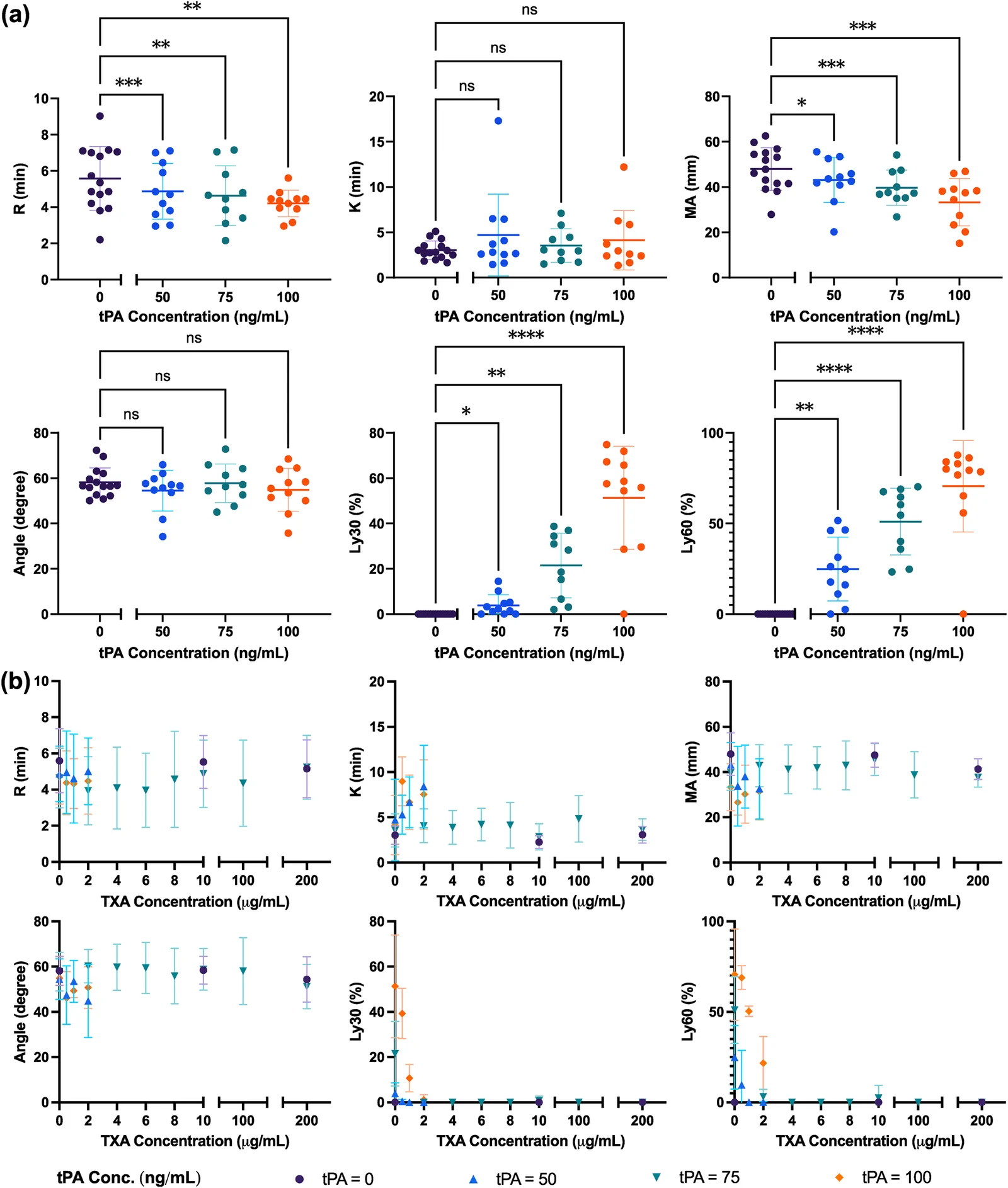
Effect of spiking tPA and TXA on six TEG parameters. **(a)** TEG parameter values (the mean of two technical replicates for each biological replicate) after varying amounts of tPA alone were spiked into whole blood. The number of donor biological replicates are: tPA at 0 ng/mL: 15; tPA at 50 ng/mL: 11; tPA at 75 ng/mL: 10; and tPA at 100 ng/mL: 11. The tPA concentrations are shown as categorical groups, and therefore, horizontal spaces between group elements do not imply numerical concentration differences. A one-way mixed effects statistical analysis was performed, followed by a Dunnett’s multiple comparisons test to compare non-zero concentrations of tPA with zero added tPA. Adjusted p values were calculated to determine statistical significance and are labeled here as: ns for p>0.05; * for 0.01<p≤0.05; ** for 0.001<p≤0.01; *** for 0.0001<p≤0.001; and **** for p≤0.0001. For each tPA level, the mean of all measured values is plotted with a horizontal line, and the standard deviation of all measured values is plotted with an error bar. **(b)** TEG parameter mean values (with standard deviation as error bars) for the tPATXA dataset. Blood samples came from fifteen donors, and each measurement had two technical replicates. No statistical analysis was performed.

TXA alone did not significantly change any TEG parameters, as the main effect of TXA is to correct hyperfibrinolysis. Therefore, we first determined the tPA concentrations for varying degrees of fibrinolysis, Figure 2a, as shown by their limited effect on R-time (R), K-time (K), maximum amplitude (MA), and alpha angle (Angle) parameters, and significant effect on the lysis parameters measured at 30 (Ly30) and 60 (Ly60) min. We performed a one-way mixed-effects statistical analysis, where the tPA dosage was the fixed effect and the donors were the random effect. We followed this with a Dunnett’s multiple comparisons test in GraphPad Prism to compare non-zero concentrations of tPA with zero added tPA. We calculated adjusted p values to determine statistical significance, and these are labeled in Figure 2a as ns for p>0.05; * for 0.01<p≤0.05; ** for 0.001<p≤0.01; *** for 0.0001<p≤0.001; and **** for p≤0.0001. At each tPA level, the mean of all measured values is plotted with a horizontal line, and the standard deviation of all measured values is plotted with an error bar.

In Figure 2b, we varied TXA concentrations from 0 to 200 μ g/mL for each color-coded tPA concentration group. At any tPA concentration, TXA had no observed effect on R, K, MA, and Angle across varying TXA concentrations, and hence no statistical analysis was performed. However, clear and consistent patterns existed with Ly30 and Ly60 parameters. For Ly30, when tPA concentrations were below 50 ng/mL, all non-zero TXA concentrations reduced lysis to zero, but when tPA concentrations were higher at 75 and 100 ng/mL, a more graded TXA effect was observed, whereby TXA concentrations below 2 μ g/mL were ineffective at mitigating lysis, while concentrations at or above 2 μ g/mL successfully eliminated observable lysis. The rationale for selecting 2 μg/mL as the boundary value of TXA concentration to suppress Ly30 at tPA concentrations above 50 ng/mL is detailed in Supplementary Section S1. For Ly60, persistent lysis was observed at all tPA concentrations of 50 ng/mL and above, whenever TXA concentrations were below 1 μ g/mL (i.e., when the TXA amount was insufficient). However, TXA concentrations of 4 μ g/mL or greater fully suppressed lysis, returning the sample to a lysis range consistent with healthy physiological conditions.

The large standard deviations observed across the data highlight biological variability among replicates, reflecting the patient-to-patient heterogeneity that is commonly encountered in clinical settings. This variability underscores the importance of personalized therapeutic strategies to tailor treatments based on individual patient responses.

### ACIT dataset

We validated model-based TEG parameter predictions using trauma patient data taken from the Activation of Coagulation and Inflammation in Trauma (ACIT) study, which has been previously described in detail [30]. This study followed 1,671 severely injured trauma patients from emergency department admission to hospital discharge or death. We used a subset of 93 patient blood coagulation profiles for this article, having an associated 254 citrated kaolin (CK) TEG assays and 122 citrated kaolin with heparinase (CKH) TEG assays. These data were excluded from model training so that they could be used solely for validation.

### Challenges and limitations of the previous model

**Figure 3:**
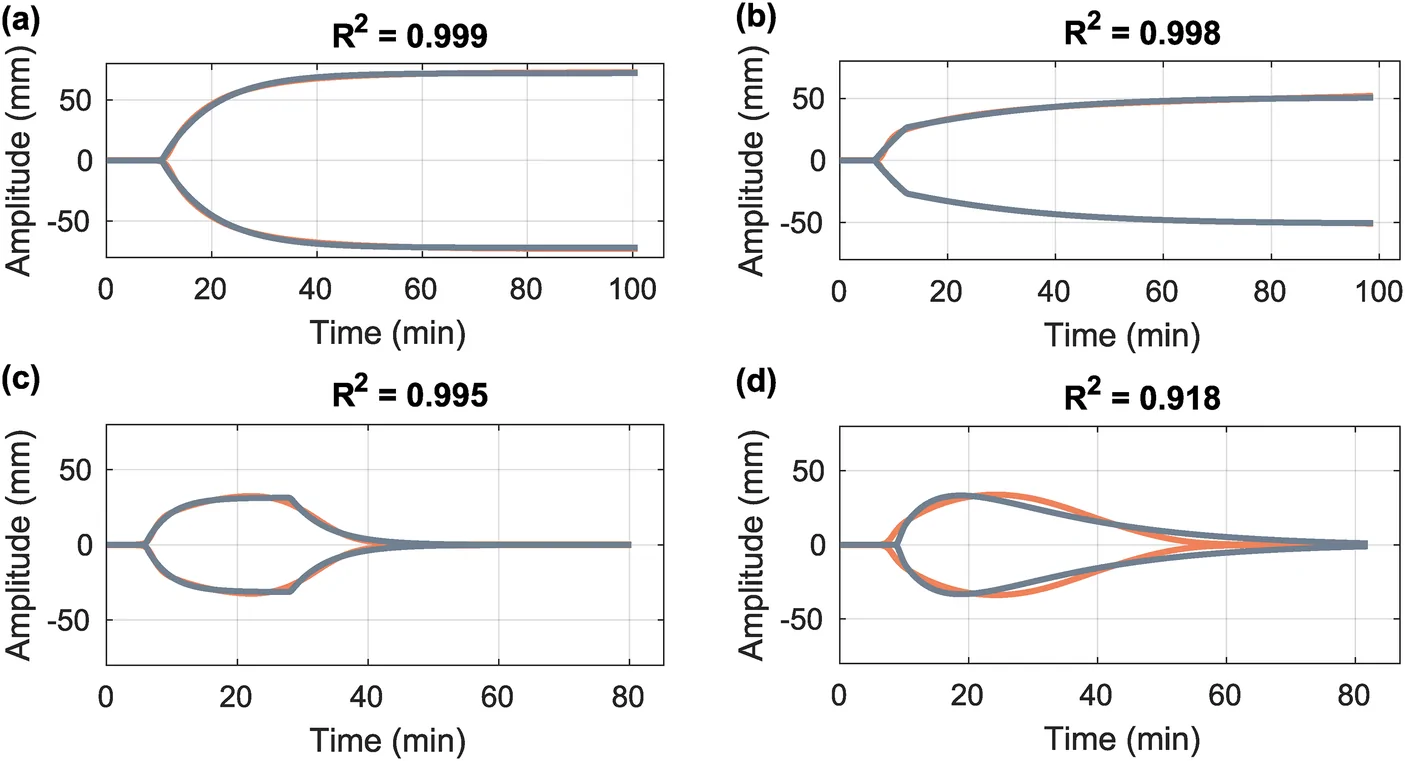
Simulated TEG profiles from our previous six-parameter model [9] in whole blood (WB) samples without and with lysis. The grey curves represent simulated TEG profiles using model 1, and the orange curves are experimental TEG profiles. Panels **(a)** and **(b)** correspond to healthy WB samples without lysis, whereas panels **(c)** and **(d)** correspond to WB samples with lysis. Discontinuities in the simulated profiles are observed in panels **(b)** and **(c)**.

The previously proposed six-parameter model 1,

$$\frac{Y(s)}{U(s)}=\frac{K_{n1}}{s(K_{\;p1}s+1)}e^{-K_{d1}s}-\frac{K_{n2}}{s(K_{\;p2}s+1)}e^{-K_{d2}s},$$ (1)

is a phenomenological model that captures the relationship between inputs and outputs, with the underlying detailed interactions modeled as a user-defined mathematical function whose parameters are determined empirically via regression analysis of experimental data. In this model, the clot strength Y(s) and the input tissue factor concentration U(s) are related through a second-order transfer function with six non-negative parameters, $K_{n1}$, $K_{\;p1}$, $K_{d1}$, $K_{n2}$, $K_{\;p2}$, and $K_{d2}$. The first term in model 1, $\frac{K_{n1}}{s(K_{\;p1}s+1)}e^{-K_{d1}s}$, approximates the coagulation process, while the second term in model 1, $-\frac{K_{n2}}{s(K_{\;p2}s+1)}e^{-K_{d2}s}$, captures fibrinolysis, or clot breakdown. The parameters $K_{d1}$ and $K_{d2}$ in the exponential terms represent the starting times of the respective processes. Given the concentrations of coagulation factors II, V, VII, VIII, IX, X, ATIII, fibrinogen, and D-dimer, the six model parameters can be quickly computed via linear mappings. Subsequently, the model 1 can generate a simulated CN-TEG profile and estimate the associated TEG parameters (R-time, K-time, MA, TMA, alpha angle, Ly30, and Ly60) within seconds. Figure 3 compares the experimental TEG profiles (orange curves) with the model-predicted counterparts (grey curves), demonstrating the model’s capability to replicate CN-TEG assay results accurately, for example, Figures 3a,b, which depicts two TEG curves that do not show any lysis.

However, model 1 still faces three critical limitations. First, as observed in Figures 3b,c, discontinuities are present due to the onset of the fibrinolysis term in model 1. While these discontinuities do not affect the clinical interpretability of the model, since clinicians primarily rely on the overall shape of the TEG profile and its associated TEG parameters to make their judgments, they pose a challenge for advanced control automation applications. In particular, the lack of smoothness violates the Lipschitz continuity condition that is required by many modern nonlinear control system methodologies, which makes it challenging to develop rigorous feedback control laws based on the model.

Second, model 1 also lacks a physical interpretation. For instance, $K_{d2}$, the onset time of fibrinolysis, takes surprisingly small values in cases where TEG profiles have no significant lysis. This parameter is designed to closely align with the time point at which the TEG curve begins to decline in amplitude, as shown by Figures 3c,d. While the TEG profile in Figure 3b has no sign of lysis, $K_{d2}$ = 12, suggesting that fibrinolysis should have begun at approximately 12 min, which is contradictory to a visual inspection of the TEG profile.

Third, the use of tissue factor as the input signal to the fibrinolysis term, $-\frac{K_{n2}}{s(K_{\;p2}s+1)}e^{-K_{d2}s}$, is counterintuitive. Acute injuries damage endothelial cells, exposing the cell interiors to the bloodstream. To stop bleeding and heal wounds, the human body undergoes two stages of hemostasis. During primary hemostasis, platelets are activated to form platelet plugs at the injury site, temporarily reducing blood loss. Stabilized blood clots are then formed to seal the wounds further during secondary hemostasis. At this stage, the exposed tissue factor from the damaged endothelial cells triggers the extrinsic coagulation cascade, substantially elevating the level of thrombin that subsequently cleaves fibrinogen to produce fibrin strands. These strands polymerize into mesh structures that stabilize the platelet plugs and produce a blood clot. On the other hand, fibrinolysis degrades blood clots to prevent excessive clotting, or dissolves clots when the injury is cured. Logically, the extent of clot degradation should be related to the amount of clot formed. In other words, fibrinolysis should depend on the output of the coagulation process. Therefore, from a modeling perspective, the fibrinolytic term should function as a scale factor that acts on the predicted clot strength. However, as shown in model 1, the fibrinolytic process is modeled as a separate summative term that shares the same input (i.e., tissue factor) as the coagulation term. This summative modeling structure challenges biological intuition and limits model 1 interpretability.

### Model formulation

We propose the following nine-parameter time-varying TEG model, represented in state-space as:

$$\begin{array}{cc} {\dot{x}}_{1}(t) & =-\frac{1}{K_{\;p1}}x_{1}(t)+\frac{1}{K_{\;p1}}\delta (t-K_{d1})u(t),\\ {\dot{x}}_{2}(t) & \;\;\;\;\;\;\;\;\;\;\;\;\;\;\;\;\;\;\;\;\;\;\;\;\;\;\;\;\;\;\;\;\;\;\;\;\;\;\;\;\;\;\;\;\;\;\;\;\;\;\;\;\;\;=K_{n1}x_{1}(t),\\ y(t) & \;\;\;\;\;\;\;\;\;\;\;\;\;\;\;\;\;\;\;\;\;\;\;\;\;\;\;\;\;\;\;\;\;\;\;\;\;\;\;\;\;\;\;\;\;\;\;\;\;\;\;=K_{s}(t)x_{2}(t), \end{array}$$ (2)

where

$$\begin{array}{rl} & K_{s}(t)=\\ & \underset{\;f_{c}(t)}{\underbrace{(1+K_{nc}(1+\tanh(K_{\;pc}t-K_{dc})))}}\underset{\;f_{f}(t)}{\underbrace{(1-K_{n2}(1+\tanh(K_{\;p2}(t-K_{d2}))))}} \end{array}.$$ (3)

In model 2, state $x_{1}(t)$ is thrombin concentration in picoMolar (pM); state $x_{2}(t)$ is the concentration of fibrin monomers (pM) produced due to the presence of thrombin; and output y(t) corresponds to the amplitude in millimeters (mm) of the strength of the blood clots. The term $\delta (t-K_{d1})$ represents a time delay by $K_{d1}$ minutes on the effect of the input tissue factor u(t) in picoMolar (pM). $K_{s}(t)$ is a time-dependent function that captures the scaling effect on blood clot strength by platelets, fibrinogen, and the formation and breakage of cross-links in fibrin strands. All nine parameters $K_{n1}$, $K_{\;p1}$, $K_{d1}$, $K_{nc}$, $K_{\;pc}$, $K_{dc}$, $K_{n2}$, $K_{\;p2}$, and $K_{d2}$ are nonnegative. Their interpretation is in Table 1.

**Table 1:** Model parameter interpretations of model 2.

Process	Model Parameters	Interpretation	Units
Coagulation	$K_{n1}$	Scaling effect on blood clot strength by platelets	1/min
	$K_{\;p1}$	Speed of clot formation	1/min
	$K_{d1}$	Time to initial clot formation	min
	$K_{nc}$	Scaling effect on blood clot strength that is not captured by $K_{n1}$	N/A
	$K_{\;pc}$	Speed of clot formation that is not captured by $K_{\;p1}$	N/A
	$K_{dc}$	Delay of the $f_{c}(t)$ function	min
Fibrinolysis	$K_{n2}$	Scaling effects on blood clot strength by fibrin-mesh degradation	N/A
	$K_{\;p2}$	Speed of clot breakage	N/A
	$K_{d2}$	Time to initial clot breakage, i.e., delay of the $f_{f}(t)$ function	min

All nine parameters are time invariant.

In general, adding model parameters will improve model performance, but adding too many parameters can cause overfitting. Compared with model 1, we introduce three additional model parameters, $K_{nc}$, $K_{\;pc}$, and $K_{dc}$, to better capture how the cross-linking between fibrin strands affects overall clot strength. The value of $f_{c}(t)$ ranges from 1 to $2K_{nc}$, where $K_{nc}$ scales the magnitude of $f_{c}(t)$, capturing the maximum strengthening effect of the fibrin cross-linking process on clot strength; $K_{\;pc}$ steers the slope of the tanh function, representing the rate of cross-link formation; and $K_{dc}$ controls the onset time of the tanh function, approximating the time when fibrin fibers begin to cross-link. Thus, we add three parameters to independently capture three missing pieces of information. If we added fewer than three parameters, we would not sufficiently capture the missing information in model 1, and if we added more than three parameters, we would overfit.

The model 2 features a modular design. The first ordinary differential equation (ODE) in model 2 is itself a highly simplified, phenomenological model of the coagulation cascade that captures thrombin generation dynamics driven by tissue factor (TF). Thus, this ODE can be replaced by a more sophisticated system of ODEs describing thrombin generation dynamics. For example, the CAT model

$$\frac{Y(s)}{U(s)}=\frac{b}{s^{3}+a_{2}s^{2}+a_{1}s+a_{0}}e^{-sT},$$ (4)

that our team developed and validated can serve as a module to incorporate CAT information into the TEG model. This phenomenological CAT model was first proposed [10] to describe the input-output relationship between TF and thrombin, approximating all biochemical interactions in Figure 1b from TF through thrombin as a third-order frequency-domain Equation 4 with five nonnegative parameters, $a_{0}$, $a_{1}$, $a_{2}$, b, and T. The CAT model 4 was converted to a state-space representation, Equations 5a–5d, in the time domain [12]. This representation can be augmented by the latter two equations in model 2, so that Equation 5e captures fibrin monomer production, and Equation 5f outputs blood clot strength. Our new combined CAT-TEG model leveraging model 2 has the following state-space representation:

$${\dot{x}}_{1}(t)=-d_{1}x_{1}(t)-\beta x_{1}(t)x_{2}(t)+\delta (t-K_{d1})u(t),$$ (5a)

$${\dot{x}}_{2}(t)=p-d_{2}x_{2}(t)-\beta x_{1}(t)x_{2}(t),$$ (5b)

$${\dot{x}}_{3}(t)=\gamma x_{1}(t)x_{2}(t)-d_{3}x_{3}(t),$$ (5c)

$${\dot{x}}_{4}(t)=kx_{3}(t)-d_{4}x_{4}(t),$$ (5d)

$${\dot{x}}_{5}(t)=K_{n1}x_{4}(t),$$ (5e)

$$y(t)=K_{s}(t)x_{5}(t),$$ (5f)

where the state vector x(t) corresponds to the concentrations of TF, factor VII, factor Xa, thrombin, and fibrin monomers, respectively. The various $d_{i}$, i = 1, 2 , 3, and 4, are nonnegative degradation rates of the respective coagulation factors; p>0 represents the production rate of factor VII; β>0 is the sequestration rate of TF and factor VII to produce TF-VIIa complexes; γ>0 is the activation rate of factor X due to TF-VIIa, and k>0 is the activation rate of thrombin induced by factor Xa.

### Model parameter evaluation

We used a custom MATLAB script with a MATLAB nonlinear least squares (trust region reflective) curve-fitting algorithm to compute model 2 parameters. Our script parses the exported files from the TEG5000 analyzer, uses the experimental TEG profile as input, and, for each TEG analysis, finds a set of model parameters to produce a simulated clotting strength trajectory that best replicates the experimental profile. As model parameters are nonnegative, the lower bounds were set to zero, while the upper bounds were set to infinity. The initial values for all states were assumed to be zero. By default, the simulation step size was set to five seconds, consistent with the sampling time of the TEG5000 analyzer. However, smaller time steps are feasible according to user needs. The Optimality Tolerance value was set to 10${}^{-9}$; the Max Function Evaluation value was set to 10${}^{6}$; the Step Tolerance value was set to 10${}^{-9}$; and the Max Iterations value was set to 5,000. No other convergence criteria or stopping conditions were used in our simulations. We emphasize here that all model parameters were computed using the curve-fitting algorithm. In future work, we will develop a nonlinear mapping to estimate these model parameters from coagulation factor levels alone, rather than through curve-fitting experimental TEG profiles.

### Performance characterization

**Figure 4:**
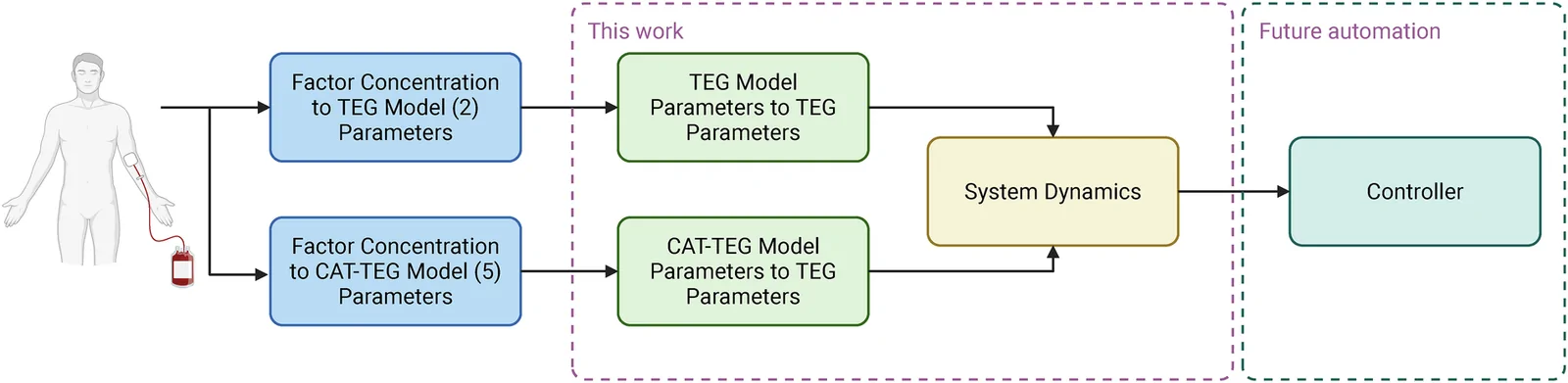
This work focuses on accurately predicting TEG parameters (R, K, MA, TMA, Angle, Ly30, and Ly60) by improving model structure. The system dynamics derived from output parameters can be used to develop control algorithms. Future automation will leverage the predicted system dynamics and existing controllers to enable closed-loop, personalized trauma care.

In this work, we aim to predict static TEG parameters rather than TEG profiles. In other words, we assume that we have access to a perfect mathematical mapping (blue blocks in Figure 4) that estimates model 2 parameters from coagulation factor levels, such that the simulated TEG profiles with the model parameters from the mapping are indistinguishable from those obtained using parameters estimated by curve-fitting experimental TEG data.

We used two metrics to characterize model performance. First, the coefficient of determination, also known as the R-squared value ($R^{2}$), quantified the fitting performance, that is, how well the simulated TEG profiles from the model replicate the experimental TEG assay. $R^{2}$ is a common metric in regression analysis, and a recent study shows that $R^{2}$ is more informative than other metrics such as mean square error (MSE) and root mean square error (RMSE) [31]. $R^{2}$ usually ranges from 0 to 1, with values closer to 1 indicating better agreement between the model and experiment, and, in our case, better model ability to capture the dominant dynamics of clot formation and degradation. For instance, an $R^{2}$ value of 0.65 suggests that approximately 35% of the variance in the data remains unexplained, likely due to unmodeled biological complexity. $R^{2}$ can be negative when the predictions made by the model are worse than those of a mean predictor.

Second, we characterized prediction performance by the percentage error between key TEG parameters (R-time, K-time, alpha angle, maximum amplitude, and time to maximum amplitude) estimated solely using the simulated TEG curves and those exported directly from the TEG5000 analyzer. For fibrinolysis time parameters Ly30 and Ly60, we used the absolute prediction error instead, as the experimental values for these parameters are zero in samples with no observable lysis. In such cases, the percentage error becomes undefined due to division by zero. Because Ly30 and Ly60 measure the percentage of clot lysis at 30 min and 60 min, respectively, after TMA, all prediction errors share the same units of percent (%).

To assess the sensitivity of the simulated TEG curves and predicted TEG parameters to variations in model parameters, we conducted a one-at-a-time (OAT) sensitivity analysis on the tPATXA dataset. For each sample in the tPATXA dataset, we treated the fitted model parameters as baselines around which we varied one model parameter at a time across a ±10% range while keeping all other model parameters fixed. We used the resultant model parameters to produce a simulated TEG curve and predicted the corresponding TEG parameters. We then compared these to the TEG parameters predicted from the baseline model parameters by computing the normalized sensitivity coefficient [32], Equation 6, using the central difference method:

$$S_{i,j}=\frac{TP_{i}(\theta_{j}+\epsilon \theta_{j})-TP_{i}(\theta_{j}-\epsilon \theta_{j})}{2\delta TP_{i_{BL}}},$$ (6)

where $TP_{i}$, i∈{1,2,…,7}, indicates the i-th TEG parameter whose baseline value is denoted as $TP_{i_{BL}}$; $\theta_{j}$, j∈{1,2,…,9}, is the j-th TEG model parameter; and ϵ=10% is the maximal relative perturbation applied to the baseline parameter value. Each sample had 7 × 9 = 63 normalized sensitivity coefficients. Since the sensitivity coefficients are largely affected by sample-specific baseline parameters and are susceptible to skewness and outliers, we used the coefficient median as the robust measure of central tendency, and the coefficient interquartile range (IQR) as the robust measure of statistical dispersion.

## Results

### Model performance

Across all tPATXA samples, model 2 achieved a higher mean $R^{2}$ value of 0.9983 with a much lower standard deviation (SD) of 0.0018 compared to model 1’s mean $R^{2}$ value of 0.9726 with a SD of 0.0275. The higher mean $R^{2}$ value indicates that our refined model has an enhanced ability to replicate experimental TEG profiles, and this improvement is consistent across all samples, as evidenced by the lower SD. Such achievement also holds when applying our new model to the validation ACIT dataset. The mean and SD values of $R^{2}$ for model 2 on this dataset were 0.9993 and 0.0007, respectively, whereas these values for the previous model 1 were 0.9848 and 0.0841.

**Figure 5:**
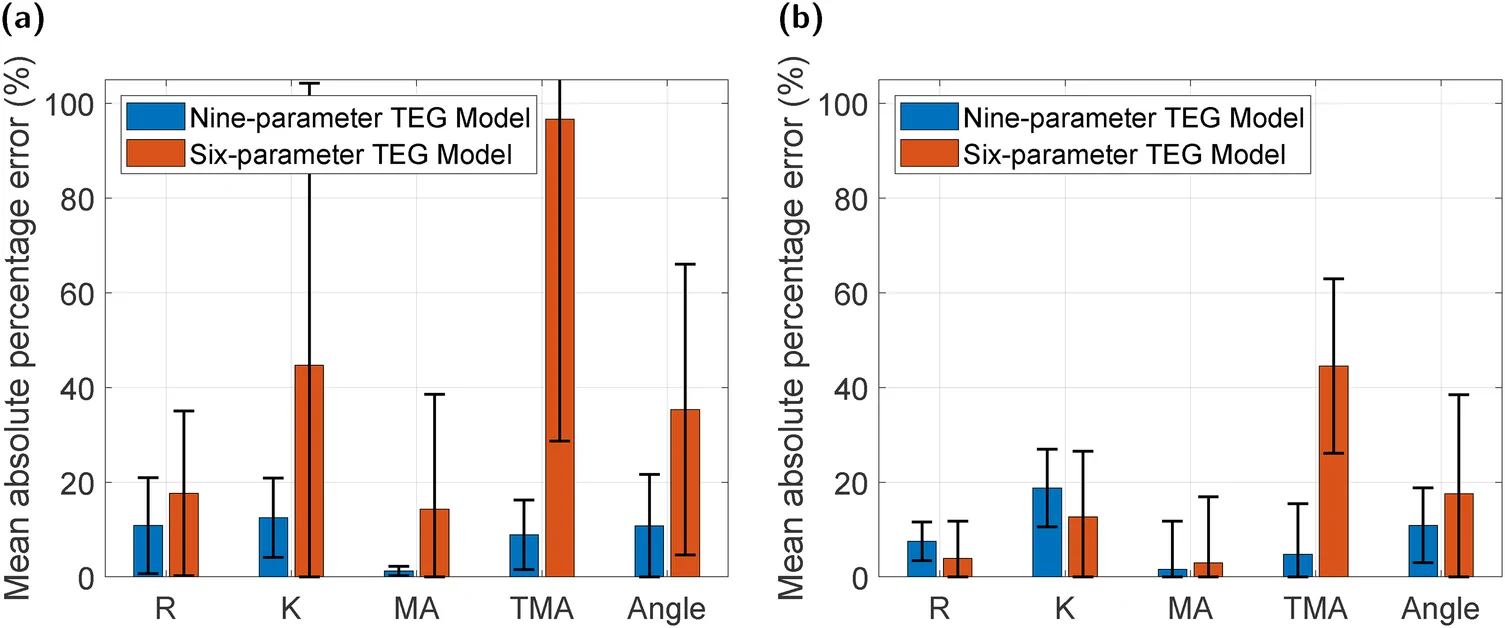
Comparison of mean absolute percentage error (MAPE) on five TEG parameters (R, K, MA, TMA, and Angle) between the nine-parameter TEG model 2 (blue bars) and the six-parameter TEG model 1 (orange bars) on **(a)** the tPATXA dataset and **(b)** the ACIT dataset. Panel **(a)** is vertically truncated such that a visual comparison between panels **(a)** and **(b)** at the same vertical scale is possible without excessive vertical data compression in panel **(b)**. For each TEG parameter, MAPE is shown as the bar height, and the error bar corresponds to the standard deviation truncated at 0 to reflect the nonnegativity of absolute percentage error (APE).

The improved predictive performance on key TEG parameters is summarized in Table 2 with the mean and SD of the prediction error for each parameter. The errors for the first five parameters are percentage-based, whereas absolute prediction errors are computed for Ly30 and Ly60, which are already in percent. Figure 5 visualizes the better predictive performance of model 2 (blue bars) compared to our previous six-parameter TEG model 1 (orange bars).

**Table 2:** Model prediction errors (mean ± SD) on key TEG parameters as given by mean absolute percentage error (MAPE, %) for R, K, MA, TMA, and Angle, and mean absolute error (MAE, %) for Ly30 and Ly60.

Dataset	tPATXA		ACIT	
Mean prediction errors	Model 1	Model 2	Model 1	Model 2
R	17.66%±17.38%	10.82%±10.13%	3.87%±7.90%	7.50%±4.06%
K	44.67%±59.55%	12.49%±8.37%	12.68%±13.88%	18.78%±8.21%
MA	14.26%±24.32%	1.23%±1.01%	2.93%±13.96%	1.59%±10.21%
TMA	96.66%±67.95%	8.87%±7.33%	44.51%±18.45%	4.80%±10.66%
Angle	35.32%±30.72%	10.79%±10.85%	17.53%±20.94%	10.87%±7.89%
Ly30	8.74%±35.45%	1.11%±2.43%	2.61%±10.74%	0.67%±2.11%
Ly60	N/A	1.71%±4.19%	N/A	2.16%±4.32%

### Sensitivity analysis

**Figure 6:**
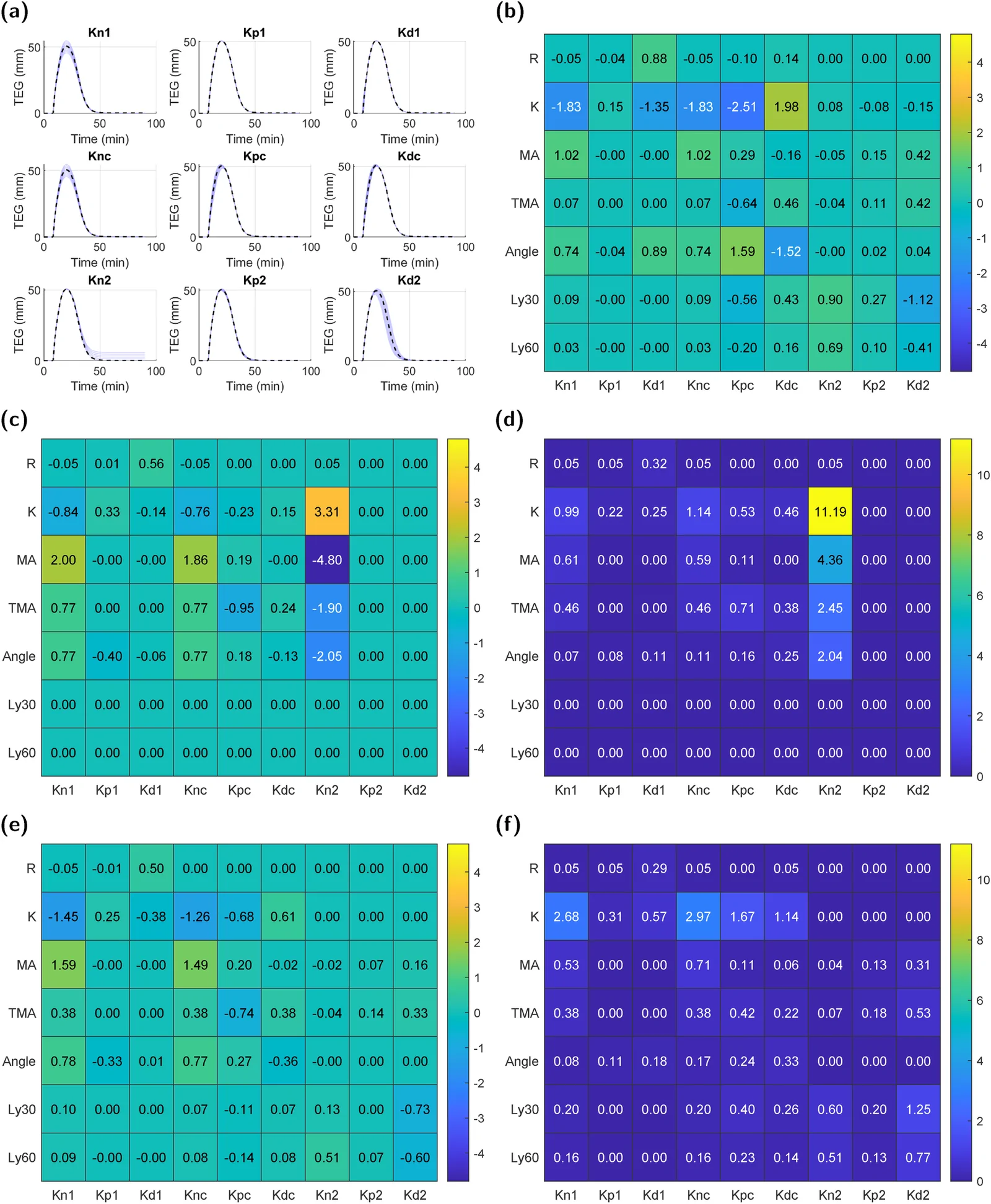
One-at-a-time (OAT) sensitivity analysis of model 2. **(a)** Effects of ±10% perturbations of individual model parameters on the predicted TEG profiles for a representative sample from the tPATXA dataset. **(b)** Heatmap of the effects of these perturbations on the corresponding TEG features of this representative sample. **(c)** Heatmap of the median signed normalized sensitivity coefficients across no-lysis samples in the tPATXA dataset. **(d)** Corresponding IQR heatmap for this no-lysis group. **(e)** Heatmap of the median signed normalized sensitivity coefficients across lysis samples in the tPATXA dataset. **(f)** Corresponding IQR heatmap for this lysis group.

Figure 6 documents the sensitivity of simulated TEG profiles and predicted TEG parameters to changes in model parameters. For illustration, we chose one representative sample from the tPATXA dataset and varied one parameter at a time while keeping the rest of the parameters fixed to their baseline values, Figure 6a. The dashed black curve is the TEG profile using baseline model parameters, and the light purple curves are TEG profiles simulated from varied model parameters. The example TEG curve shape changes with variations in $K_{n1}$, $K_{nc}$, $K_{\;pc}$, $K_{dc}$, $K_{n2}$, and $K_{d2}$, and does not change with variations in $K_{\;p1}$, $K_{d1}$, and $K_{\;p2}$. For this representative sample, the heatmap in Figure 6b quantifies how model parameter perturbations (x-axis) affect static TEG parameters (y-axis). The numerical values in the heatmap cells are the signed normalized sensitivity coefficients. These indicate the approximate percentage change of the corresponding TEG parameters resulting from a 10% change in a model parameter. Figure 6c extends Figure 6b, showing the medians of the signed normalized sensitivity coefficients for all samples in the tPATXA dataset without lysis, i.e., those samples that had a zero value for Ly30 and Ly60 (n = 146). Figure 6d shows the corresponding IQRs for these samples. We separated out samples without and with lysis when ascertaining sensitivity because the main motivation for this work is to capture lysis. Figure 6e is a heatmap of the medians of the signed normalized sensitivity coefficients for all samples in the tPATXA dataset with lysis (n = 130), and Figure 6f has the corresponding IQRs.

### Robustness to different TEG assays and TIC types

The improved model performance is independent of the type of TEG test: CN-TEG, CK-TEG, or CKH-TEG. Since clinicians may prefer one type of TEG assay over another, our TEG model must remain valid and accurate regardless of the type of assay used. Table 2 confirms that model 2 provides accurate estimates of TEG parameters for different types of TEG tests in the ACIT dataset.

**Figure 7:**
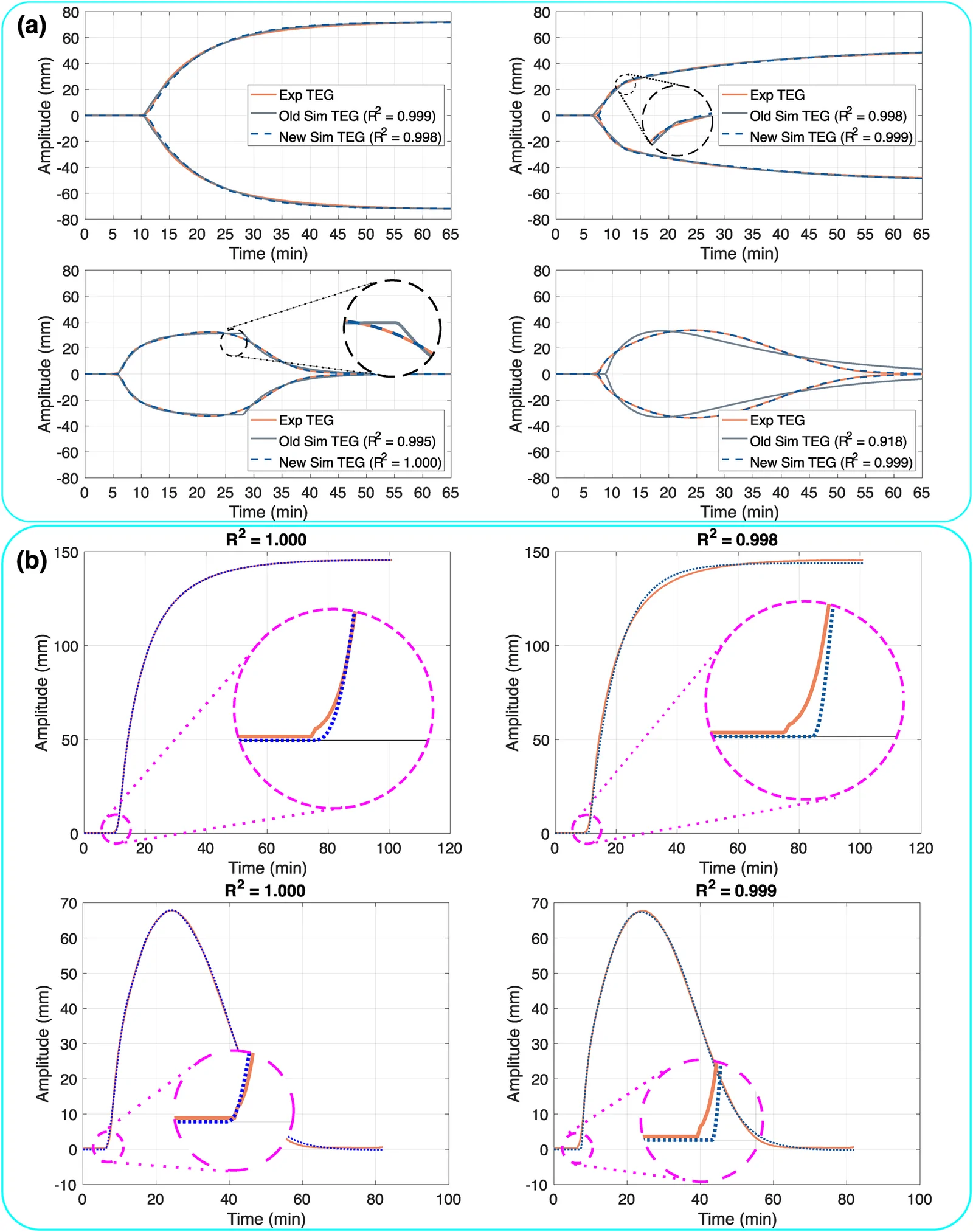
Model fitting performance comparison. **(a)** Experimental TEG profiles (orange) are compared with simulations from model 1 (gray) and model 2 (dark blue). **(b)** Comparison between the infused CAT-TEG model 5 (left column) and model 2 (right column). While both models replicate experimental data well, the CAT-TEG model achieves marginally improved fits, particularly in clot formation dynamics, as highlighted by the magnified regions.

Figure 7a depicts how our updated model addresses the limitations of the previous TEG model. First, the zoomed-in regions demonstrate that model 2 produces smooth TEG curves, without any discontinuities. Next, the values of $K_{d2}$ using the updated model do not have counterintuitive interpretations. In the top-right plot of Figure 7a, model 1 gives $K_{d2}=12.00$ for a sample without lysis, which is biologically implausible. In contrast, the updated model yields $K_{d2}=225$, a value exceeding the assay duration, thereby ensuring that the fibrinolytic term remains inactive and contributes nothing to the overall profile. Finally, the updated model shows significantly improved fitting performance; the $R^{2}$ value in the bottom right plot of Figure 7a increases from 0.918 to 0.999 by switching from model 1 to model 2, resulting in closer alignment of the overall curve shape. This is a critical correction, as the original model’s predicted TEG profile leads to an inaccurate physiological interpretation: delayed clot initiation followed by rapid clot formation. In contrast, the updated model reveals a pattern characterized by delayed and slow clot formation. Moreover, the updated model produces simulated curves that almost perfectly overlap with the experimental data, demonstrating its robustness across both normal and hyperfibrinolytic cases.

### Integrated CAT-TEG model performance

Figure 7b demonstrates the modularity of our updated TEG model. The plots in Figure 7b have not been rescaled to match the display style of the TEG analyzer. This intentional choice makes the visual discrepancies between the model predictions and the experimental data more obvious. Comparing the left two plots using model 5 with the right two plots using model 2, despite similar $R^{2}$ values, the CAT-infused model 5 can better replicate the experimental TEG data. As highlighted by the dashed circles, the simulated curves in the left two plots are more closely aligned with the experimental TEG profiles at the time when clots initiate. As R, K, and Angle are defined based on these turning points, a small misalignment between the experimental and the simulated TEG profiles leads to large prediction errors in TEG parameters.

## Discussion

Hyperfibrinolysis is a strong indicator of early mortality in current trauma care. Early detection and targeted treatments that are tailored to each patient are essential to reduce this mortality. To facilitate detection, a fast and accurate method for assessing patient coagulation profiles is desired. Such a method will not only allow clinicians to diagnose hyperfibrinolysis quickly but also provide them with frequent updates on patient blood clotting status, enabling them to adjust treatment promptly. Common coagulation assays are too time-consuming, forcing clinicians to follow pre-established, standardized protocols that avoid the worst treatment outcomes rather than target the best ones. Model-based *in silico* experiments can rapidly make predictions, thus serving as a faster alternative to experimental assays in time-critical situations. Previously, we had developed a six-parameter model to replace thromboelastography (TEG) assays. However, this model faced three limitations: discontinuities in TEG profiles, counterintuitive biological interpretations of model parameters, and inconsistent predictive performance ($R^{2}$ values) of hyperfibrinolytic samples.

In this work, we assessed a nine-parameter, time-varying TEG model that addressed the limitations of its six-parameter predecessor. Our updated model generates continuous TEG curves that align nearly perfectly and consistently with experimental profiles. Across all 310 samples in our new experimentally-generated tPATXA dataset, our updated model achieved an average $R^{2}$ of 0.9983, a 2.64% improvement over the previous model average $R^{2}$ of 0.9726. More notably, the variance of $R^{2}$ decreased from $7.5625\times 10^{-4}$ to $3.24\times 10^{-6}$, a reduction of 99.57%. Similar improvements were seen in the validation clinical ACIT dataset, where the mean $R^{2}$ increased by 1.47% and the variance reduced by 99.99%, from $7.0728\times 10^{-3}$ to $4.9\times 10^{-7}$. This large reduction in variance implies substantially more consistent fitting performance across all datasets, which is crucial in clinical settings where predictability and reliability across patient populations are essential. Because static TEG parameters (R, K, MA, TMA, Angle, Ly30, and Ly60) are derived from a TEG profile, having a simulated curve that is indistinguishable from the experimental one means that TEG parameters estimated from the simulation should, in theory, be identical to their experimental counterparts. Compared to readings from the TEG5000 analyzer, our model achieves mean percentage prediction errors of 1%–13% for R, K, MA, TMA, and Angle, and mean absolute prediction errors of 0.7% and 2.2% for Ly30 and Ly60, respectively. Compared with our previous TEG model 1, refined model 2 achieves substantially improved accuracy and consistency in predicting TEG parameters. As Figure 5a shows, all five blue bars are much shorter than the orange bars, with much narrower error bars. Although model 1 achieves lower mean prediction errors on R-time and K-time in Figure 5b, model 2 generally performs better as the error bars are much tighter and the mean prediction errors on TMA and Angle are much smaller than those of model 1. Model 2 is also superior in Ly30 predictions, as evidenced by Table 2.

**Figure 8:**
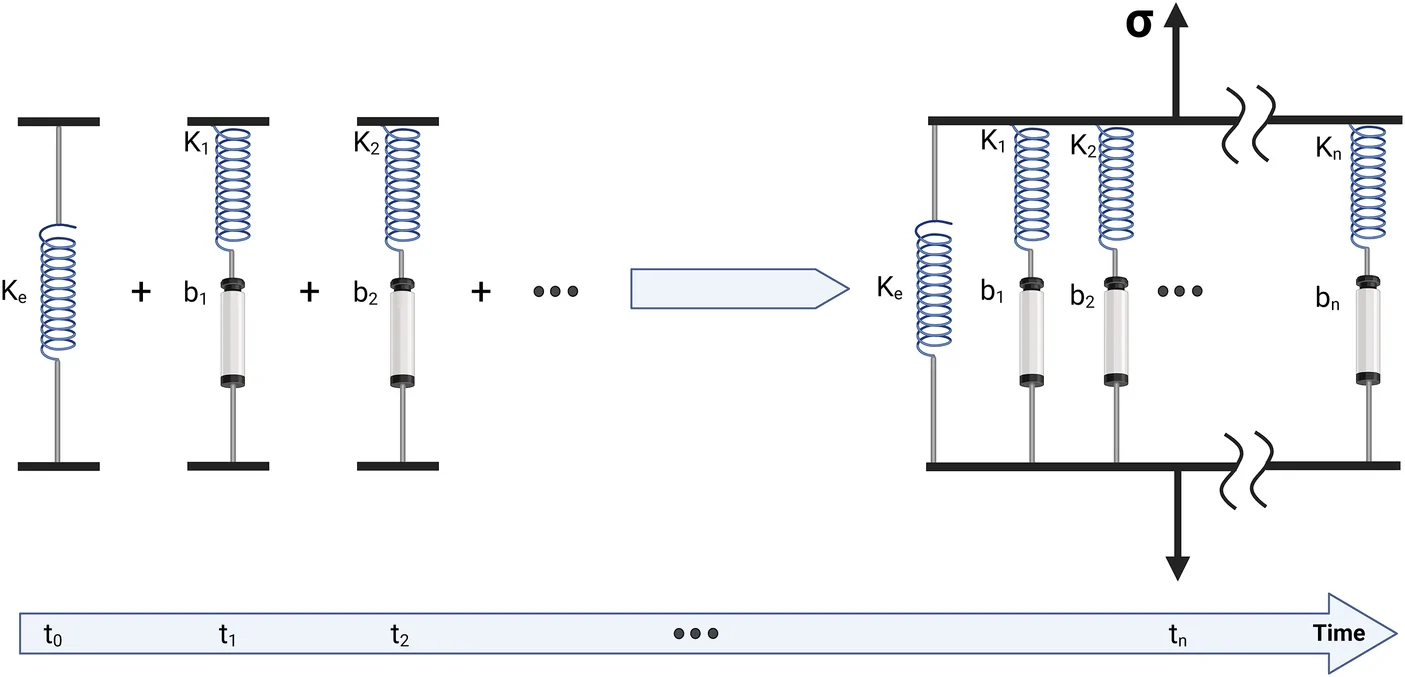
An illustration of the generalized time-varying Maxwell model. Each pair of a purely viscous damper and a purely elastic spring connected in series is a Maxwell element that mimics a fibrin strand. The concatenation of Maxwell elements over time approximates the cross-linking process between fibrin strands. This process is captured by $f_{c}(t)$.

The improved continuity and accuracy of predicted TEG curves are largely attributable to two new time-dependent sigmoidal functions, $f_{c}(t)$ and $f_{f}(t)$, which better describe the cross-linking behavior of individual fibrin strands. We can ascribe a mechanical interpretation to these functions by building upon our original model 1’s physical interpretation that used the generalized Maxwell-Wiechert model of viscoelasticity [9], where a Maxwell element consists of a spring and a damper connected in series. In this work, we consider each fibrin strand as a Maxwell element, Figure 8, and $f_{c}(t)$ can be interpreted as gradually connecting these Maxwell elements in parallel until saturation. Biologically, this process resembles the formation of the fibrin mesh structure over time during coagulation. In the other direction, $f_{f}(t)$ approximates the breakage of these parallel-connected Maxwell elements, analogous to the degradation of fibrin strands during the fibrinolysis process.

Our TEG models conform to the notion that thrombin acts as a catalyst in the fibrin production process [33]. In model 1, the delayed first-order low-pass filter, $e^{-K_{d1}s}\frac{1}{K_{\;p1}s+1}$, corresponds to the velocity profile of the TEG curve and approximates thrombin-generation kinetics, suggesting that thrombin generation affects the production rate of fibrin monomers. The integrator $\frac{K_{n1}}{s}$ predicts how many fibrin strands will be generated during the coagulation phase. In the Maxwell-material analogy of Figure 8, this corresponds to predicting how many pairs of spring-damper structures will be produced. However, the behavior of fibrin cross-linking or the parallel concatenations of the spring-damper structures is not captured by $e^{-K_{d1}s}\frac{1}{s(K_{\;p1}s+1)}$. This helps explain the inconsistent fitting performance of model 1 across TEG samples, especially in hyperfibrinolytic samples. The sigmoidal function $f_{c}(t)$ in model 2 suggests that the generation of fibrin cross-links occurs as a Hill function, which is commonly seen in biological dynamics to describe binding [34]. Similarly, the sigmoidal function $f_{f}(t)$ in model 2 suggests that fibrin-mesh degradation occurs as a Hill function. While our mechanical analogies provide biologically intuitive insights, further experimental validation is needed to determine whether these sigmoidal behaviors accurately reflect the underlying physiology.

To quantify model predictive behavior in response to perturbations of model parameters, we performed a one-at-a-time (OAT) sensitivity analysis using the tPATXA dataset and visualized the results via heatmaps in Figure 6. We found that the median of the sensitivity coefficients associated with $K_{n1}$ and $K_{nc}$ are similar to each other. This is because these two model parameters dominate the peak value of a TEG curve. Such similarity in the sensitivity coefficients can also be explained using model 2. In Equation 3, when $K_{nc}\gg 1$, $f_{c}(t)\approx K_{nc}(1+\tanh(K_{\;pc}t-K_{dc}))$. Consequently, $y(t)\approx K_{n1}K_{nc}(1+\tanh(K_{\;pc}t-K_{dc}))f_{f}(t)\int_{t_{0}}^{t}x_{1}(t)dt$, suggesting that the peak value as well as the TEG parameters that relate to the peak are determined by $K_{n1}K_{nc}$. Since this is true only when $K_{nc}\gg 1$, the $K_{n1}$ and $K_{nc}$ columns in Figures 6c–f are similar but not identical.

We determined prediction sensitivity on samples without and with lysis. For samples without lysis, Figures 6c,d suggest that the TEG parameters K, MA, TMA, and Angle are sensitive to model parameter $K_{n2}$, with large IQRs. This implication is counterintuitive because $K_{n2}$ is a fibrinolytic parameter. Since the samples do not have observable lysis, the sensitivity coefficients for these four TEG parameters to $K_{n2}$ should be close to zero. One possible explanation for this counterintuitive result relates to our design of $f_{f}(t)$, where $K_{dc}$ acts as a “switch”: when fibrinolysis does not occur in a sample, the fitting algorithm sets $K_{dc}$ to a large value, thereby delaying the onset of the $f_{f}(t)$ function to a time that exceeds the duration of a 90-min CN-TEG assay. Since we do not hard-code the values of the fibrinolytic parameters, $K_{nc}$ can be assigned any arbitrary value when $K_{dc}$ is large. Depending on these values, perturbations of $K_{nc}$ may introduce fibrinolysis, thereby affecting the four TEG parameters. This explains why the magnitudes of both the median and the IQR of the sensitivity coefficients in the corresponding entries of Figures 6c,d are large. However, in absolute terms, these magnitudes are much less than 20%, a rule-of-thumb goal predictive error in mechanical systems [35] that is considered restrictive for biological systems due to their innate variability. Therefore, our determined model prediction sensitivity is not a concern.

**Figure 9:**
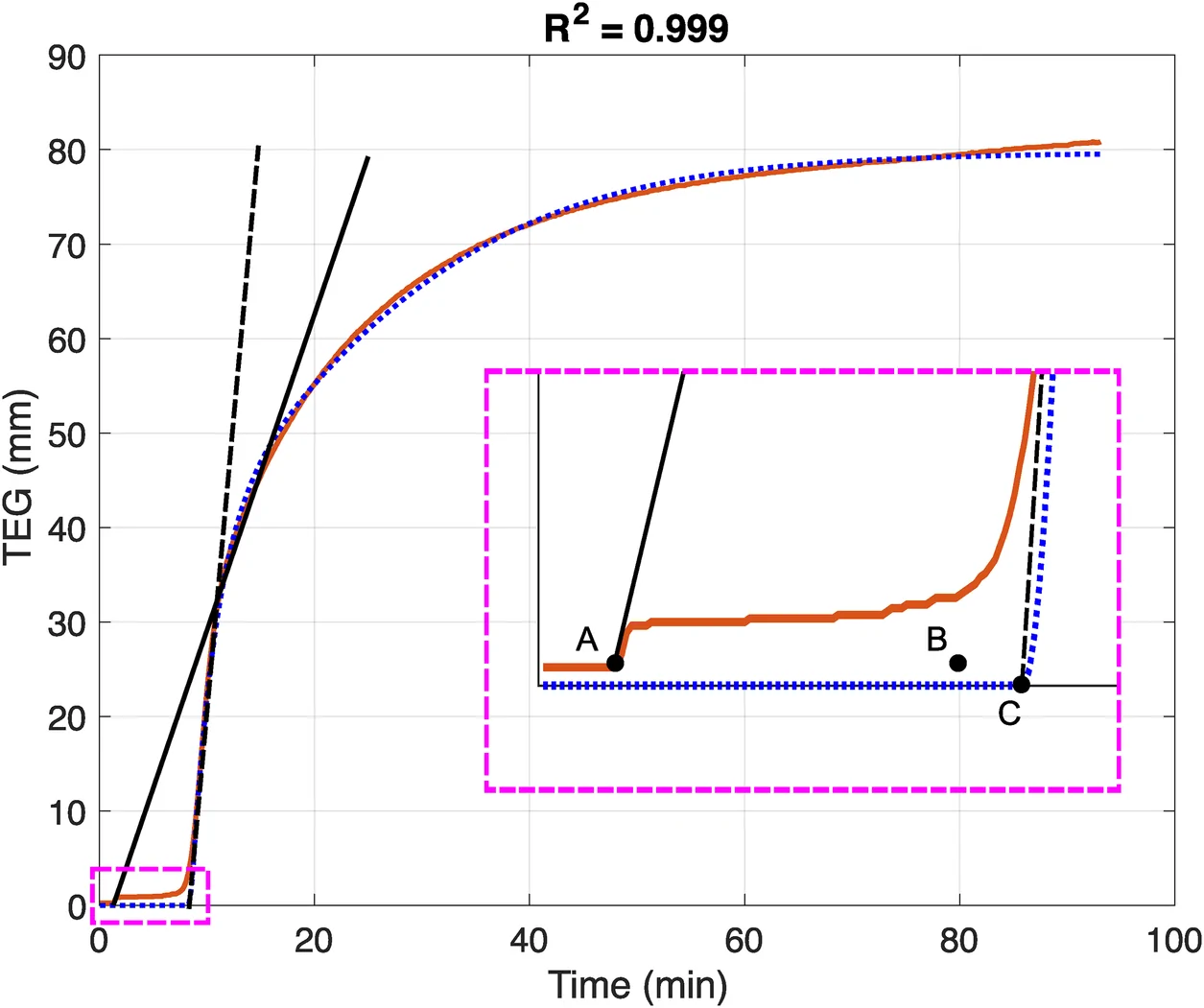
Human error in an experimental TEG assay (orange solid curve), and robustness of the model-predicted TEG profile (blue-dotted curve) to reject human error. The alpha angle is the tangential line passing through the split plot (experimental TEG assay: solid black line; simulated TEG assay: dashed black line). Point B is the true split point of the experimental TEG profile, assuming no human errors. Point A is the actual split point, and point C is the split point on the simulated TEG profile.

The TEG parameters R-time, K-time, and alpha angle are more sensitive to human error than other parameters of the TEG trace, whereas our model-simulated profile is robust to such error. For example, in Figure 9, human error introduces an initial bump on the experimental TEG curve (highlighted in the pink box). Because the alpha angle is defined as a tangential line of the curve passing through the split point, this bump shifts the actual split point A forward relative to the true split point B, resulting in an underestimated alpha angle. The simulated profile does not contain such an error, and thus its split point C lies much closer to point B, yielding a more accurate estimate of the alpha angle. In this example, the experimental alpha angle is $23.6^{\circ }$, whereas the predicted value is $49.16^{\circ }$, causing a 108.31% prediction error. In a technical replicate of this sample without human error, the experimental and predicted alpha angles are $52.6^{\circ }$ and $53.97^{\circ }$, respectively. These results suggest that our model provides accurate estimates of TEG parameters whether or not human error is present in the experimental TEG profiles. Our findings indicate that, in clinical settings, the overall shape of TEG profiles is more reliable than the derived parameters. Human error affects only a small local region of the curve that is too minor to alter the global profile, but is sufficient to yield large errors in TEG parameters.

The strong predictive performance of our refined phenomenological model of viscoelastic clot formation and lysis suggests that our model has the potential to replace traditional experimental assays. Our model’s ability to generate instant predictions provides a foundation for implementing control-theoretic tools to actively regulate clot strength, and the combined CAT-TEG model 5 enables precise adjustment of blood clot strength using existing control-theoretic tools. Our previously developed controllers dedicated to the nonlinear CAT model 5a-5d allowed the thrombin concentration obtained from Equation 5d to track a given trajectory [11, 13]. Since the state whose dynamics are in Equation 5d affects the rate of fibrin production in Equation 5e, our previously developed controllers should allow us to regulate the blood clot strength precisely, and we will explore this control application in future work.

## Code availability

The MATLAB model is available at: https://github.com/SYBORGS-Lab/Refined-Viscoelastic-Clot-Model The algorithm code is available for personal use. The code is subject to the original patent application number PCT/US2024/026140 (international) and 19/478,527 (U.S.), copyright: Copyright 2023 University of Florida Research Foundation, Inc. All commercial rights reserved.

## Data Availability

The datasets presented in this study can be found in online repositories. The names of the repository/repositories and accession number(s) can be found in the article/Supplementary Material.
