# Supplementary material for: A refined phenomenological model of viscoelastic clot formation and lysis in trauma-induced coagulopathy
Source: Front Cardiovasc Med. 2026 Sep 3;13:1816085. doi: 10.3389/fcvm.2026.1816085 (PMC13582509; doi:10.3389/fcvm.2026.1816085)
Supplement: Supplementary file 1 [file Supplementaryfile1.pdf]

Supplementary Section S1

TXA Concentration to Remove Lysis at 30 Minutes for tPA Above 50 ng/mL

Initially, a standard *in vitro* concentration of 75 ng/mL tPA[36] was used to induce clot lysis. Fibrinolysis countering TXA concentrations of 10 and 200 μg/mL were selected based on prior literature[37], while 0.075 μg/mL was chosen to match the tPA concentration. Ly30 values were analyzed with GraphPad Prism using a one-way analysis of variance (ANOVA) in Supplementary Figure 1(a), and a two-way ANOVA in Supplementary Figure 1(b). Post hoc comparisons were made against the zero TXA condition using a Dunnett's multiple comparisons test. We calculated adjusted p values to determine statistical significance, and these are labeled in Supplementary Figure 1 as ns for $p > 0.05$; * for $0.01 < p \leq 0.05$; ** for $0.001 < p \leq 0.01$; *** for $0.0001 < p \leq 0.001$; and **** for $p \leq 0.0001$. At each TXA level, the mean of all measured values is plotted with a horizontal line, and the standard deviation of all measured values is plotted with an error bar.

Supplementary Figure 1(a) shows that Ly30 values did not significantly differ between 0 and 0.075 μg/mL applied TXA, whereas both 10 and 200 μg/mL applied TXA effectively abolished clot lysis within 30 minutes. We then refined the TXA concentration range to determine the minimum therapeutic dose to eliminate lysis, Ly30 plot in Figure 2(b). Three biological replicates were spiked with 75 ng/mL tPA and TXA at 2, 4, 6, 8, 10, 50, 100, 150 μg/mL. High variability and a limited number of biological replicates resulted in a lack of statistical significance between zero TXA and non-zero TXA conditions, but the data trends suggested that TXA concentrations $\geq$2 μg/mL consistently suppressed Ly30 values to zero.

Consequently, the TXA range was refined once more, and two tPA concentrations on either side of 75 ng/mL, 50 ng/mL and 100 ng/mL, were tested, Supplementary Figure 1(b). At a tPA concentration of 50 ng/mL, minimal clot lysis was observed, and no significant changes in Ly30 were detected across the tested TXA doses. These findings are consistent with Figure 2(b) observations that 50 ng/mL tPA induces limited lysis, insufficient to classify as hyperfibrinolytic. Thus, even low TXA concentrations are sufficient to reduce lysis to baseline in this condition. In

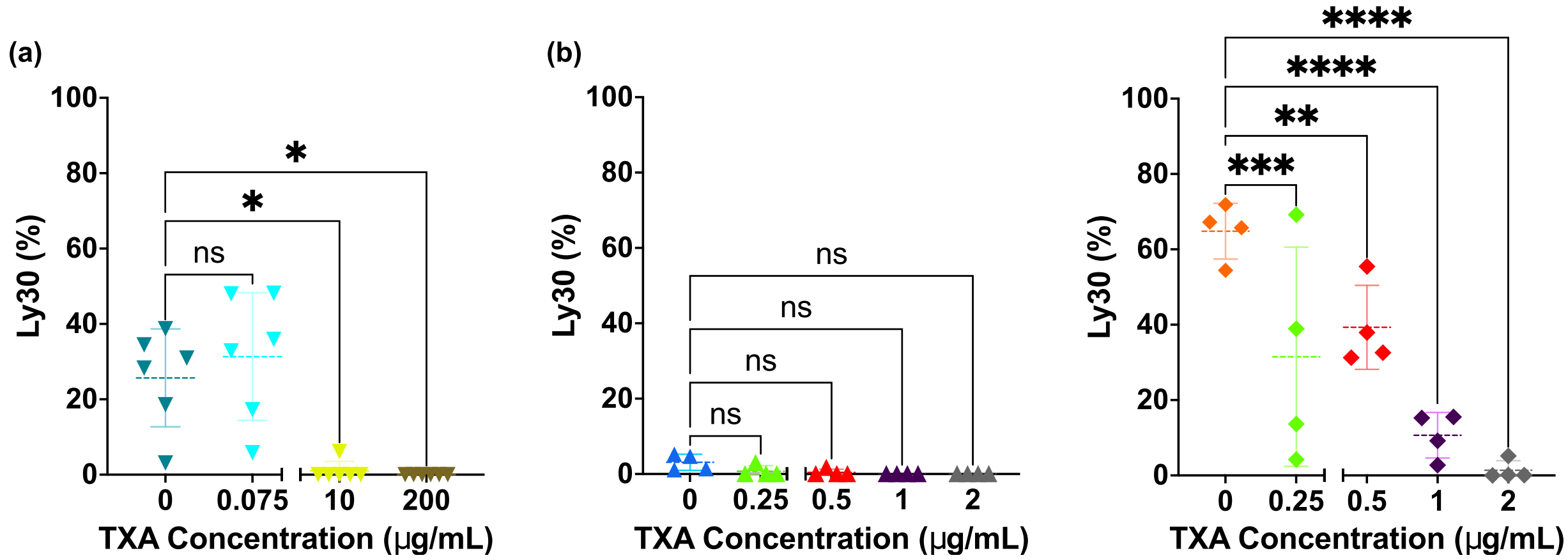

Supplementary Figure 1: Effect of TXA concentration on Ly30 (%) at different tPA concentrations. In both panels, TXA concentrations are shown as categorical groups, and therefore, horizontal spaces between group elements do not imply numerical concentration differences. **(a)** Whole blood clot lysis after 30 minutes for tPA at 75 ng/mL concentration and varying concentrations of TXA. Data were obtained from six donor blood samples, and the mean of two technical replicates per donor is shown. A one-way ANOVA followed by a Dunnett's multiple comparisons test was performed. Adjusted p values were calculated to determine statistical significance and are labeled here as ns for $p > 0.05$ and * for $p \leq 0.05$. **(b)** Whole blood clot lysis measured after 30 min at two tPA concentrations, 50 ng/mL (left) and 100 ng/mL (right), across a refined range of TXA concentrations. For each TXA concentration within each tPA condition, four donor blood samples were tested, and the mean of two technical replicates per donor is shown. A two-way ANOVA followed by a Dunnett's multiple comparisons test was performed. Adjusted p values were calculated to determine statistical significance and are labeled here as: ns for $p > 0.05$; * for $0.01 < p \leq 0.05$; ** for $0.001 < p \leq 0.01$; *** for $0.0001 < p \leq 0.001$; and **** for $p \leq 0.0001$. For each TXA level, the mean of all measured values is plotted with a horizontal line, and the standard deviation of all measured values is plotted with an error bar.

contrast, for a tPA concentration of 100 ng/mL, every tested TXA concentration produced a statistically significant reduction in Ly30 compared to the no-TXA control. While all TXA doses reduced fibrinolysis, only concentrations ≥2 μg/mL brought Ly30 values below the hyperfibrinolysis threshold, suggesting that 2 μg/mL is the minimum dose required to fully counteract the fibrinolytic activity of 100 ng/mL tPA.

Supplementary References

36. Moore HB, Moore EE, Gonzalez E, Wiener G, Chapman MP, Dzieciatkowska M, et al. Plasma is the physiologic buffer of tissue plasminogen activator-mediated fibrinolysis: rationale for plasma-first resuscitation after life-threatening hemorrhage. Journal of the American College of Surgeons. 2015;220(5):872-9.

37. Godier A, Parmar K, Manandhar K, Hunt BJ. An in vitro study of the effects of t-PA and tranexamic acid on whole blood coagulation and fibrinolysis. Journal of Clinical Pathology. 2017;70(2):154-61.
